# S100A4-neutralizing antibody suppresses spontaneous tumor progression, pre-metastatic niche formation and alters T-cell polarization balance

**DOI:** 10.1186/s12885-015-1034-2

**Published:** 2015-02-12

**Authors:** Birgitte Grum-Schwensen, Jörg Klingelhöfer, Mette Beck, Charlotte Menné Bonefeld, Petra Hamerlik, Per Guldberg, Mariam Grigorian, Eugene Lukanidin, Noona Ambartsumian

**Affiliations:** 1 Institute of Neuroscience and Pharmacology, Faculty of Health Sciences, Copenhagen University, 2200 Copenhagen, Denmark; 2 Danish Cancer Society Research Center, 2100 Copenhagen, Denmark; 3 Institute of International Health, Immunology and Microbiology, Faculty of Health Sciences, Copenhagen University, 2200 Copenhagen, Denmark

## Abstract

**Background:**

The tumor microenvironment plays a determinative role in stimulating tumor progression and metastasis. Notably, tumor-stroma signals affect the pattern of infiltrated immune cells and the profile of tumor-released cytokines. Among the known molecules that are engaged in stimulating the metastatic spread of tumor cells is the S100A4 protein. S100A4 is known as an inducer of inflammatory processes and has been shown to attract T-cells to the primary tumor and to the pre-metastatic niche. The present study aims to examine the immunomodulatory role of S100A4 *in vivo* and *in vitro* and assess the mode of action of 6B12, a S100A4 neutralizing antibody.

**Methods:**

The therapeutic effect of the 6B12 antibody was evaluated in two different mouse models. First, in a model of spontaneous breast cancer we assessed the dynamics of tumor growth and metastasis. Second, in a model of metastatic niche formation we determined the expression of metastatic niche markers. The levels of cytokine expression were assessed using antibody as well as PCR arrays and the results confirmed by qRT-PCR and ELISA. T-cell phenotyping and *in vitro* differentiation analyses were performed by flow cytometry.

**Results:**

We show that the S100A4 protein alters the expression of transcription factor and signal transduction pathway genes involved in the T-cell lineage differentiation. T-cells challenged with S100A4 demonstrated reduced proportion of Th1-polarized cells shifting the Th1/Th2 balance towards the Th2 pro-tumorigenic phenotype. The 6B12 antibody restored the Th1/Th2 balance. Furthermore, we provide evidence that the 6B12 antibody deploys its anti-metastatic effect, by suppressing the attraction of T-cells to the site of primary tumor and pre-metastatic niche. This was associated with delayed primary tumor growth, decreased vessel density and inhibition of metastases.

**Conclusion:**

The S100A4 blocking antibody (6B12) reduces tumor growth and metastasis in a model of spontaneous breast cancer. The 6B12 antibody treatment inhibits T cell accumulation at the primary and pre-metastatic tumor sites. The 6B12 antibody acts as an immunomodulatory agent and thus supports the view that the 6B12 antibody is a promising therapeutic candidate to fight cancer.

**Electronic supplementary material:**

The online version of this article (doi:10.1186/s12885-015-1034-2) contains supplementary material, which is available to authorized users.

## Background

In recent years it has become evident that the tumor microenvironment is deeply engaged in determining the metastatic fate of the tumor [1]. Many components of the stroma can influence the metastatic spread of tumor cells by modulating the molecular network in the tumor milieu. Similarly, the microenvironment of secondary organs, where metastases develop, plays a crucial role. Molecular changes in the microenvironment of secondary organs contribute to the formation of pre-metastatic niches, the future location where cancer cells will reside, proliferate and develop metastases [2,3].

Therapeutic targeting of cells comprising the tumor stroma by ablation was suggested as a novel and efficient way to combat cancer [4]. Immune cells that represent a substantial component of the stroma in many solid human tumors exhibit a remarkable dichotomy between tumor-suppressing and tumor-promoting functions. From a therapeutic prospective, this plasticity can be used to educate immune cells to become tumor-suppressing, which is a more advantageous strategy than simply eradicating immune stroma cells, as was suggested earlier [5]. For example, it has been shown that tumor-associated macrophages, educated to be pro-tumorigenic by T-cell-produced cytokines, can be re-educated to exhibit tumor suppressing functions [6].

Similar to macrophages, lymphocytes also play a dual role in the tumor microenvironment by regulating both pro- and anti-tumor immunity [7].

Among the numerous molecules of the tumor microenvironment that play causal roles in metastatic spread of cancer cells is the S100A4, which belongs to the S100 family of small Ca-binding proteins. This group of proteins is characterized by both intra- and extra-cellular activity. S100A4 is expressed in many human cancers, and is correlated with poor prognosis and an elevated incidence of metastasis [8,9]. By using transgenic and knockout mouse models stroma-cell derived S100A4 was shown to have a causal role in tumor progression [10-15]. It has been suggested that it modulates the microenvironment, both at the site of the primary tumor and the pre-metastatic niche [13,15,16].

Tumor-associated fibroblasts are one of the sources of extracellular S100A4 in tumors [12,15]. S100A4-positive fibroblasts produce VEGF-A and tenascin-C, which in turn contribute to generating a pro-metastatic environment [15]. Pro-tumorigenic signal transduction pathways as well as the production of proteases and cytokines from various cell types are activated by S100A4 [17-20]. Furthermore, it has been shown that S100A4 acts as an angiogenic factor, as well as attracting T-cells to the site of the growing tumor and pre-metastatic lungs [11,13,20,21]. Unfortunately, receptors mediating extracellular functions of S100A4 remain elusive. Several receptors have been suggested by the research community including RAGE, TLR-4 and EGFR, pointing to the possibility of multiple-receptor interaction of S100A4 at the cell surface [22].

Taking into account its pivotal role in metastasis, S100A4 was suggested as a potential target for a novel cancer therapy. For examples, the anti-inflammatory drug sulindac, which inhibits S100A4 transcription, effectively suppressed colon cancer metastasis [23]. Recently, we have shown that the S100A4 function-blocking antibody (6B12) suppressed metastasis formation in mice grafted with metastatic mammary cancer cells. Furthermore, this study suggested that, the anti-S100A4 antibody decreased metastatic burden by blocking the attraction of T-cells [24]. *In vitro*, S100A4 was chemo-attractive for T-cells and modified the pattern of cytokines produced by these cells [13].

Based on the above, we propose that S100A4 might alter the T-cell balance in the tumor microenvironment and thereby promote cancer metastasis. We further suggest that blocking S100A4 activity can reinstate the “normal” T-cell balance and by this suppress the metastasis.

Here we show that S100A4 -challenged T-cells showed reduced amount of Th1-polarized cells by this altering the Th1/Th2 polarization balance. The Th1/Th2 balance is restored by the S100A4 neutralizing antibody. By implementing two different mouse models we demonstrate that the S100A4 function blocking antibody suppresses spontaneous tumor progression and pre-metastatic niche formation which correlates with suppression of T-cell accumulation both at the site of primary tumor and in pre-metastatic lungs.

## Methods

### Reagents

RPMI 1640, PBS and FCS were from (Gibco Life Technologies). Protease inhibitors were from (Roche). Recombinant mouse IL2 was from (Miltenyi Biotech). Mouse IgG and rabbit IgG were from (Sigma-Aldrich, USA). Isolation of the oligomeric form of the S100A4 protein and the S100A4 mutant (G47W) was described earlier [25,26]. Isolation and characterization of 6B12 anti-S100A4 mouse monoclonal antibody was described in [24].

### Animal experiments

#### Ethical considerations

All mouse experiments were performed according with the charter of fundamental animal rights of the European Union (20007C364/01, Dec 7 2000). Permission to work with mouse tumor models and breeding of genetically modified animals has been granted to Noona Ambartsumian by the Dyreforsøgstilsynet, Fødevarestyrelsen. (Danish Agency for Animal Experiments, Food Administration) (license 2013-15-2934-00864/ACHOV). All animals were maintained according to the FELASA guidelines. All animals were health checked daily. Tumors were measured at least twice a week and mice were sacrificed when tumors reach 1 cm^3^ or if they have any clinical signs of illness or distress due to the tumor burden. Detailed description of experimental procedures was done in accordance with the ARRIVE guidelines and could be found in Additional file 1.

### PyMT mouse model

Virgin female PyMT mice (Polyoma-middle T spontaneous metastatic mammary cancer model) of A/Sn genetic background were used for the experiments. Breeding and genotyping was performed as described earlier [13]. 6-week-old PyMT female mice were injected either with a loading dose (7.5 mg/kg) of 6B12 (n = 20), or IgG control (n = 20), intra-peritoneal. Injections of antibodies were repeated three times a week. 5 animals of each experimental group were sacrificed at age of 12 weeks. For the rest animals were sacrificed and processed as described earlier when the biggest tumor reached 1 cm^3^ [13].

### Pre-metastatic assay

CSML100 metastatic mouse mammary carcinoma cells (1×10^6^) [27] were injected subcutaneously into S100A4(-/-) mice of A/Sn genetic background [11] followed by intravenous injection of 2.5×10^5^ S100A4(+/+) or S100A4(-/-) mouse embryonic fibroblasts (MEFs). Experiment was repeated twice.

For the analysis of the antibody effect, CSML100 cells (1×10^6^) were injected subcutaneously followed by intravenous injection of 2.5×10^5^ S100A4(+/+) or S100A4(-/-) mouse embryonic fibroblasts (MEFs) mixed with either 100 μg of 6B12, or IgG control. Then the mice were injected with a loading dose (7.5 mg/kg) of 6B12, or IgG control intra-peritoneally three times a week. Injections of MEFs mixed with antibodies were repeated three times with a one-week interval. Animals were sacrificed when the tumor reaches 5-6 mm in diameter (pre-metastatic phase) [13]. Experiment was repeated twice.

### Cytokine antibody and PCR array analyses

Pre-metastatic lungs were incubated for 2 hours in PBS at 37°C. Conditioned media (CM) from five individual lung cultures were combined in equal ratio and applied to RayBio Mouse Cytokine Antibody Arrays 3 and 4, allowing simultaneous analysis of 96 cytokines (RayBiotech Inc.). The assay was performed according to the manufacturer’s instructions.

The mouse Th1-Th2-Th3 RT Profiler™ PCR Array (SABiosciences) was used to determine the relative expression of 84 genes related to CD4^+^ T-helper cells, according to the manufacturer’s instructions. RayBio Mouse Cytokine Antibody Array 1 for detecting 22 cytokines was used to test the cytokine profile in the T-cell CM.

### Immunohistochemistry

Formalin-fixed paraffin embedded tissue sections were stained with antibodies against CD3 (rabbit polyclonal, catalogue #ab5690; Abcam), mouse α-smooth muscle actin (clone 1A4; Sigma-Aldrich), anti-Fibronectin (FN) (clone AB-10; Neomarkers), anti-CD31 (clone MEC 13.3) and anti-CD45 (clone 30 F11), both from BD Biosciences according to the manufacturer’s protocols.

Corresponding secondary horse-radish peroxidase-conjugated antibodies (DAKO, Glostrup, Denmark) were used, followed by incubation with chromogenic substrate 3,3′-diaminobenzidine (DAKO).

For double staining, secondary antibodies coupled to Alexa Fluor 488 or 568 (1:1500) were purchased from Molecular Probes. Sections were examined by means of confocal microscopy on a LSM 510 (Carl Zeiss Inc).

The blood vessel density was determined by quantifying CD31+ capillaries in 3-4 fields from two different sections of the tumor (magnification, ×200).

Quantification of CD3+ T-cells in tumors from 12-week-old PyMT mice and in pre-metastatic lungs was performed as described [13].

### Protein expression analysis

Proteins isolated from the pre-metastatic lungs were resolved by SDS-PAGE. FN expression was analyzed using a standard Western-blot procedure with anti-FN (DAKO) antibodies. Mouse anti-tubulin-α (clone AA13; Sigma-Aldrich) was used as a loading control. MultiGauge software was used for data analysis (Fujifilm).

To test the Jak-Stat signalling pathway activation, purified T-cells were starved in RPMI 1640 for 3 h and stimulated for 5 and 10 minutes with S100A4 protein (1 μg/ml), or mixed with 6B12 antibody (6 μg/ml).

Cell lysates were prepared in the presence of protease- and phosphatase-inhibitors. Activation of the Jak3/Stat3 signalling pathway was analysed using a standard Western blot procedure with phospho-Janus Kinase 3 (Jak3; Tyr^980^/Tyr^981^, clone D44E3), phospho-Signal Transducer and Activator of Transcription 3 (Stat3; Tyr^705^, clone D3A7) and Jak3 (clone D7B12) and Stat3 (clone D3Z2G), antibodies (Cell Signaling Technology). Each experiment was reproduced 3 times using independent primary T-cell isolations.

### RNA sample preparation and quantitative real-time polymerase chain reaction (qRT-PCR)

T-cells purified as described in [13] were stimulated with S100A4 (1 μg/ml) for 19 h in presence of 10 μg/ml Polymyxin B (Invitrogen). Total RNA was isolated using a NucleoSpin® TriPrep kit (Macherey-Nagel). First-strand cDNA synthesis was performed using Super-Script III RT according to the manufacturer instructions. qRT-PCR was performed using a LightCycler 2.0 instrument (Roche Applied Science, USA). The expression level relative to the housekeeping GAPDH gene, as a control, was calculated. The PCR analysis was repeated 3 times using independent primary T-cell isolation. The primers used in this work are presented in Table 1.

**Table 1:** **List of primers used for analysis**

Gene	Forward primer	Reverse primer
FN	5′-TGCCGCAACTACTGTGAT-3′	5′GAATCCTGGGCTGGAGTA--3′
G-CSF	5′-CAGATCACCCAGAATCCAT-3′	5′-CTCTCGTCCTGACCATAGTG-3′
Jak3	5′-TGGCCACTGAGGACTTCTCT-3′	5′-GGATGGCACTGGTCAAATCT-3′
IL6	5′-ACAAGAAAGACAAAGCCAGA-3′	5′-TAGCCACTCCTTCTGTGACT-3′
GATA3	5′-CTGGAGGAGGAACGCTAATG-3′	5′-GTTGAAGGAGCTGCTCTTGG-3′
IL10	5′-TCTCCCCTGTGAAAATAAGA-3′	5′-TCCAGCAGACTCAATACACA-3′
Tyk2	5′-ATCCGTTTGTACAGGCCAAG-3′	5′-GCTGTGTGATGGGGAACTTT-3′
CD40	5′-GGCTTCGGGTTAAGAAGGAG-3′	5′-GCAGGGATGACAGACGGTAT-3′
CTLA4	5′-GGATCCTTGTCGCAGTTAGC-3′	5′-AAACGGCCTTTCAGTTGATG-3′
TGFβ	5′-TGCGCTTGCAGAGATTAAAA-3′	5′-CGTCAAAAGACAGCCACTCA-3′
GAPDH	5′-TCATCCCTGCATCCACTG-3′	5′-TAGGAACACGGAAGGCCA-3′

### T-cell phenotyping

Spleen, thymus and inguinal and brachial lymph nodes were dissected from 8-week-old A/Sn S100A4(+/+) and S100A4(-/-) mice [11]. Single-cell suspensions were prepared from the organs. Cells were counted, adjusted to 1 × 10^7^cell/ml and plated in 96-well plates (100 μl/well). Distribution and activation status of T/B cells was assessed using a cocktail of primary conjugated antibodies: anti-CD4 (clone RM4-5), anti-CD8a (clone 53-6.7), anti-CD19 (clone ID3), anti-TCRαβ (clone H57-597), anti-CD25 (clone PC61), anti-CD44 (clone IM7), or anti-CD62L (clone MEL-14) (BD Biosciences). Cells were incubated with the antibodies (1:100) in ice-cold PBS containing 2% FCS and 0.1% NaN_3_ for 30 min on ice. Data acquisition and analysis were performed on aFACSCalibur (BD Biosciences) using FlowJo software (Tree Star).

### T cell isolation and in vitro differentiation

Primary T-cells were isolated from 3-4 mouse spleens by negative selection on magnetic bids using the Pan T cell Isolation Kit II (Miltenyi Biotech), see Grum-Schwensen *et al*. for details [13]. Purified T-cells were maintained in RPMI for 3 or 6 days with anti-CD3 or a combination of anti-CD3 and anti-CD28 antibodies, coupled to MACSibeads (Miltenyi Biotec) plus 10 ng/ml recombinant IL2 as described in [28].

Activated T-cells were stimulated with S100A4 protein (1 μg/ml) or S100A4 protein mixed with 6B12 antibody (6 μg/ml). Before fixation, PMA/Ionomycin and Golgistop™ (BD Biosciences) were added for 5 hours. Cells were washed with PBS and Fixable Viability Stain 450 (BD Biosciences) was added to discriminate between viable and dead cells.

Cells were fixed using the Cytofix/Cytoperm™ kit (BD Biosciences) and stained with the mouse Th1/Th2/Th17 phenotyping kit (BD Biosciences) containing antibodies against CD4, IL17A, IFNγ and IL4 according to the manufacturer’s instructions. Data acquisition and analysis were performed on a FACSVerse (BD Biosciences) using FlowJo software (Tree Star). All experiments were repeated 3-5 times.

### Viability and proliferation assay

Cell viability and proliferation were measured by the LDH (Roche) and CyQuant® cell proliferation assay kit according to the manufacturer’s instructions.

### ELISA assay

The concentration of the cytokines IL2, IL4, IFNγ and IL4 in the CM from the T-cell cultures was measured using the Mouse Th1/Th2 ELISA assay (eBioscience) according to the manufacturer’s instructions. The experiment was performed twice.

### Statistical analysis

The confidence level was calculated using paired or unpaired Student’s *t* test, depending on the content of the experiment, using GraphPad Prism software. Data is shown as mean ± SEM.

## Results

### S100A4 activates T-cell differentiation signalling pathways

As we have shown earlier, the treatment of T-cells with S100A4 protein *in vitro* led to the production of distinct cytokines [13]. This opened up the possibility that similar changes occurred in the pre-metastatic lungs *in vivo*. To test this idea, we implemented the recently developed mouse model of pre-metastatic niche formation [13].

Briefly, the metastatic CSML100 mammary carcinoma cells displayed a suppressed ability to form metastases when grafted to S100A4(-/-) mice. However, they could be primed to metastasize by intra-venous administration of S100A4 expressing fibroblasts (S100A4(+/+)MEFs), but not by S100A4(-/-) MEFs. In this model we defined the ‘metastatic stage’ as the point when tumor-bearing mice contained pulmonary metastases, and the ‘pre-metastatic stage’ when tumors were small (5-6 mm) and lungs contained only solitary cancer cells. Notably, S100A4(+/+), but not S100A4(−/−) MEFs, stimulated the accumulation of T-cells in the lung parenchyma, suggesting a potential role of T-cells in the formation of a pro-metastatic milieu [13].

To further characterize the changes that occur in the pre-metastatic lungs we compared the cytokines released in pre-metastatic lung organ cultures from S100A4(+/+) MEF versus S100A4(-/-) MEF boosted tumor-bearing mice using a cytokine antibody array. This experiment revealed altered expression of a number of cytokines including elevated levels of G-CSF and eotaxin-2 (Table 2). We also detected an increase in the level of IL4 and IL9 and a decrease in the level of IFNγ and IL1 compared to control. These might reflect a modulation in the pattern of T-cells in pre-metastatic lungs. Also noted was the change in expression of cytokines not involved in T-cell differentiation, suggesting the complexity of changes that occur in pre-metastatic lungs.

**Table 2:** **Relative expression of cytokines in the pre-metastatic lung organ cultures isolated from S100A4(-/-) tumor-bearing mice**

	Injected cells		
	CSML100 (s/c)	CSML100(s/c)	CSML100 (s/c)
	No MEFs	S100A4(+/+) MEF(i/v)	S100A4(-/-) MEF(i/v)
**eotaxin-2**	**1**	**2,21**	**1,56**
**G-CSF**	**1**	**2,17**	**1,04**
**IL6**	**1**	**1,99**	**2,18**
**fractalkin**	**1**	**1,83**	**1,64**
**IL9**	**1**	**1,79**	**1,61**
**Lix**	**1**	**0,72**	**1,58**
**IL4**	**1**	**1,89**	**1,50**
**M-CSF**	**1**	**1,52**	**1,25**
**KC (GRO-alpha)**	**1**	**1,44**	**1,19**
**MIP-1-gamma**	**1**	**1,22**	**1,42**
**SDF-1alpha**	**1**	**2,11**	**1,39**
**IGFBP6**	**1**	**1,43**	**2,03**
**IL1-alpha**	**1**	**0,67**	**1,31**
**IL1-beta**	**1**	**0,41**	**1,60**
**L-selectin**	**1**	**0,59**	**0,95**
**lymphotactin**	**1**	**2,15**	**1,65**
**MCP1**	**1**	**1,62**	**1,26**
**PF4**	**1**	**0,77**	**2,31**
**IFN-gamma**	**1**	**0,77**	**0,82**
**ctack**	**1**	**0,68**	**1,42**

**Figure 1:**
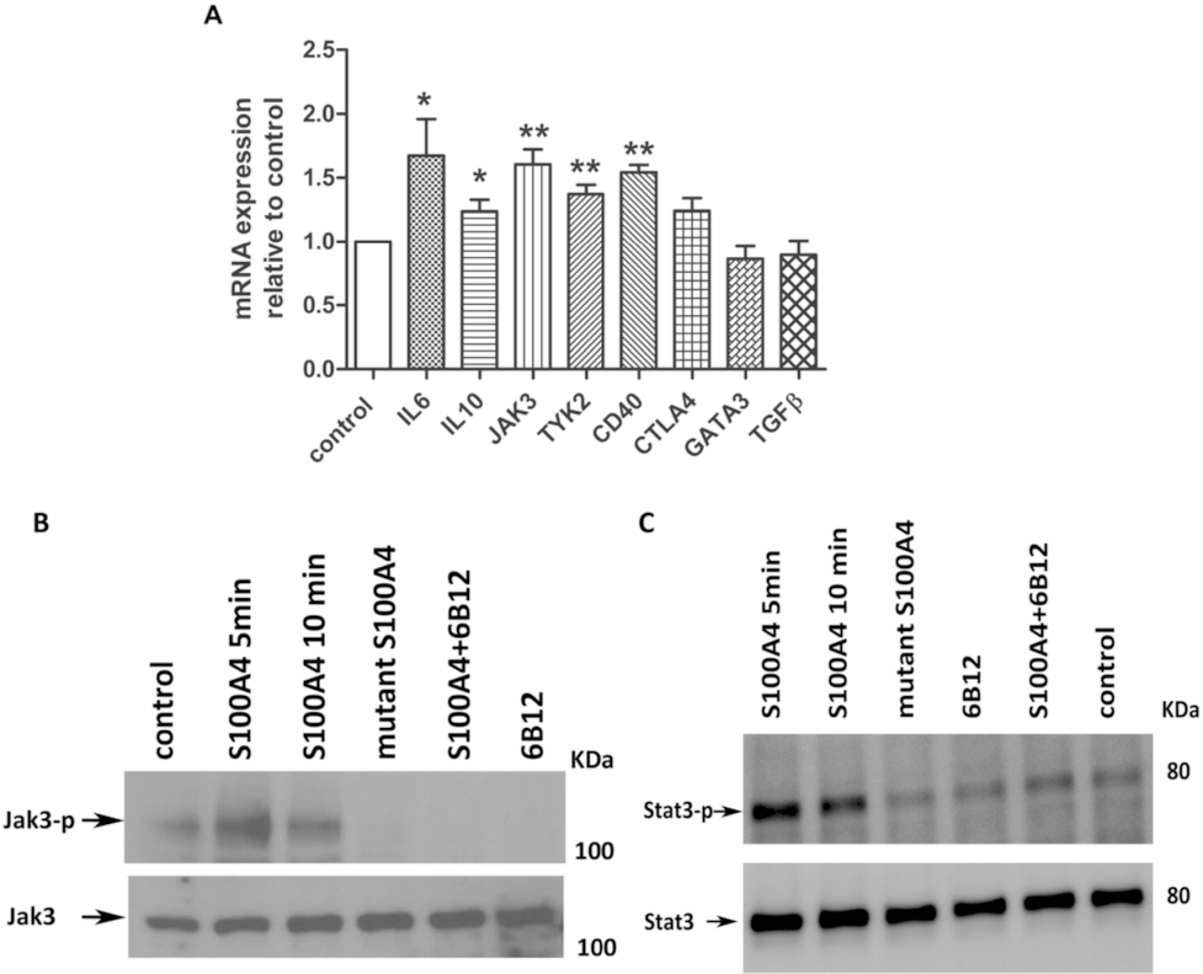
**S100A4 activates T-cell differentiation pathways*in vitro.*(A)** Relative expression of IL6, IL10, Jak3, Tyk2, CD40, CTLA4, GATA3 and TGF-β as determined by qRT-PCR of RNA isolated from T-cells treated with the S100A4 protein. *, *P* < 0.05; **, *P* < 0.01. **(B)** Phosphorylation of Jak3 in T-cells treated either with S100A4 protein, mutant S100A4 protein, S100A4 protein mixed with 6B12, or 6B12 alone. T-cells were stimulated for 5 and 10 min. Western blot analysis with anti-phospho-Jak3 and anti-Jak3. **(C)** Phosphorylation of Stat3 in T-cells treated with S100A4 protein, mutant S100A4 protein, S100A4 protein mixed with 6B12, or 6B12 alone. T-cells were stimulated for 5 and 10 min. Western blot analysis with anti-phospho-Stat3 and anti-Stat3 antibodies.

We further performed gene expression profiling of S100A4-stimulated primary T-cells that revealed changes in the expression of genes responsible for the differentiation of CD4^+^ T-cells (Additional file 2: Table S1). The differential expression of selected genes was validated by independent experiments using qRT-PCR (Figure 1A). While S100A4 had little effect on the transcription of GATA3 and TGF-β, the transcription of IL6, IL10, Jak3, Tyk2, CD40 and CTLA4 was substantially increased in S100A4-treated primary T-cells. To further validate the results of gene expression profiling, we examined the level of selected cytokines produced by S100A4-stimulated T-cells. This analysis confirmed the previously observed increase in IL6, IL2 and IL10 levels. We also detected an increase in the production of IL9, IL17, IL13 and decrease in IFNγ. The complete list of differentially expressed cytokines is presented in the Additional file 2: Table S2.

The resulting data also demonstrated that S100A4 modulates the expression of transcription factor and signal transduction pathway genes involved in determining the fate of T-helper lineage. Alteration of T-cell polarization could be predicted based on the up-regulation of Jak3, Stat1, Socs3, Tyk2 as well as down-regulation of Tbx21 (t-bet), GATA3, Socs1 and Socs5 [29].

We next tested whether S100A4 could activate the Jak/Stat pathway which is known for its implication in the lineage differentiation of T-cells [30]. Western blot analyses of Jak3 and Stat3 phosphorylation of T-cell protein extracts showed strong activation of Jak3 and modest activation of Stat3 upon S100A4 treatment, whereas the S100A4 mutant protein did not activate the phosphorylation (Figure 1B and C). Moreover, the S100A4-neutralizing 6B12 antibody efficiently blocked phosphorylation of both, Jak3 and Stat3 (Figure 1B and C). These results opened the possibility that S100A4 affects T-cell differentiation via activation of the Jak/Stat pathway.

Analysis of cytokine antibody and gene expression arrays directed us to study the effect of S100A4 on the T-cell differentiation.

### S100A4 alters the Th1/Th2 balance of differentiating T-cells by reducing the amount of Th1-polarized cells

**Figure 2:**
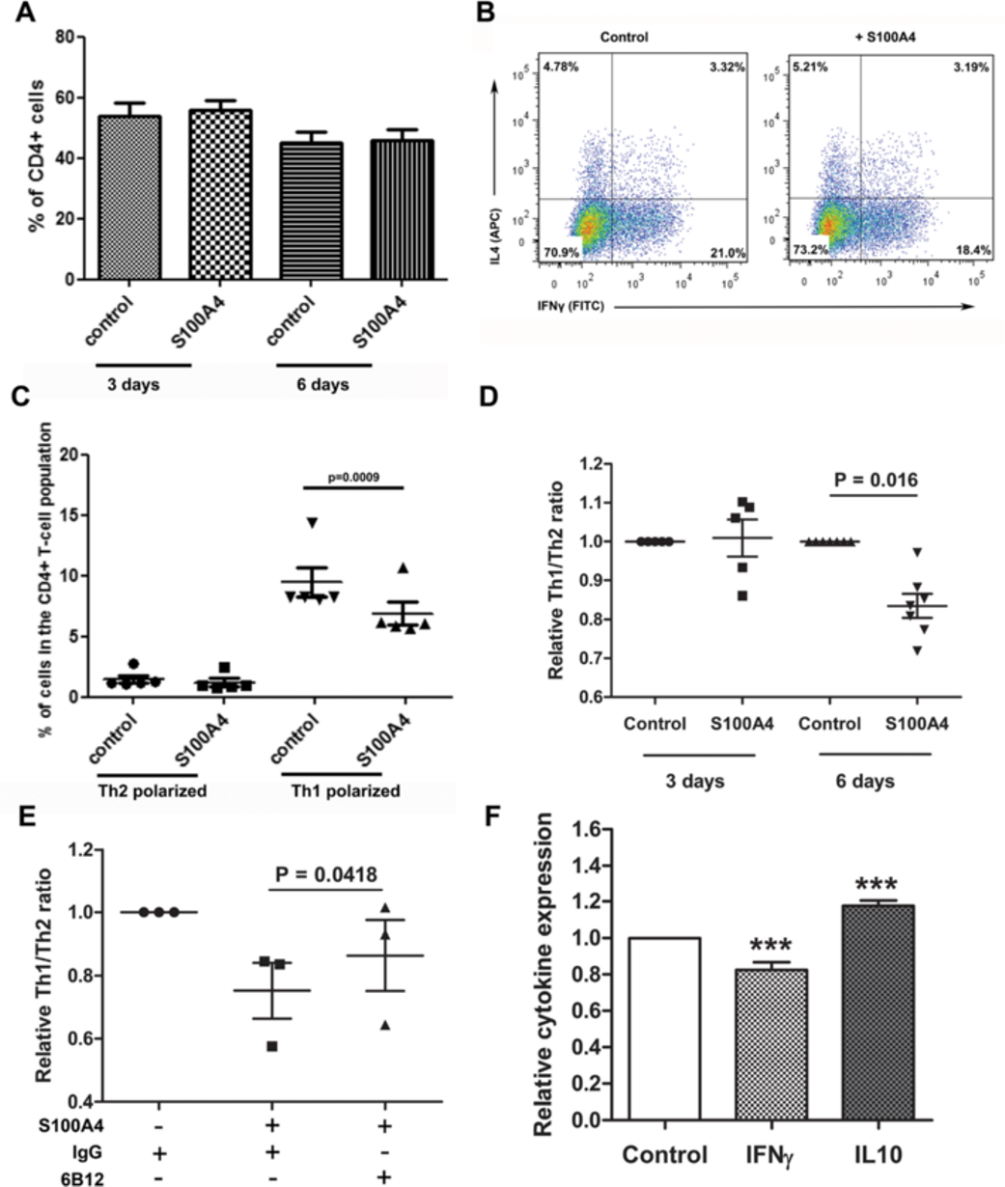
**S100A4 shifts the Th1/Th2 balance of differentiating T-cells in vitro. (A)** The percentage of CD4^+^ T-cells in S100A4-treated and control T-cell populations at 3 and 6 days. **(B)** Representative pictures of the flow cytometry analysis of primary Th1/Th2 polarized CD4^+^ T-cells. Primary T-cells were expanded with or without S100A4 protein for 6 days. **(C)** The percentage of Th1-polarized CD4^+^ T-cells in the S100A4-treated and control populations. **(D)** The relative ratio of Th1/Th2 polarized T-cells after stimulation with S100A4 protein for 3 and 6 days. **(E)** The relative ratio of Th1/Th2 cells after stimulation of T-cells for 6 days with the S100A4 protein or with S100A4 protein mixed with 6B12. **(F)** Determination of IFNγ and IL10 levels in CM from T-cells expanded with or without S100A4 protein for 6 days by Sandwich ELISA. Data are in triplicate. ***P < 0.001.

We next assessed the long-term effect of S100A4 on T-cells. Isolated primary T-cells were primed to proliferate by CD3 and propagated for 3 and 6 days with or without S100A4. The percentage of live cells (data not shown) as well as the percentage of CD4^+^ in both cultures at 3 and 6 days was similar (Figure 2A), suggesting that S100A4 did not alter the population of CD4^+^ T-cells. The CD4^+^ T-cell fraction was further analysed to establish changes in the T-helper cell populations. The Th1/Th2 ratio at 3 days and Th2/Th17 ratio at 3 and 6 days remained unchanged by S100A4-treatment (data not shown). In contrast, at 6-days the percentage of Th1-polarized cells in S100A4-treated cultures was significantly reduced while the fraction of Th2-polarized cells remained unaffected (Figure 2B and C). Priming S100A4-treated T-cell with CD3/CD28/IL2, to stimulate production of Th1-polarized cells, did not reverse the situation (Additional file 2: Figure S1). Relative Th1/Th2 ratios showed a significant shift towards prevalence of Th2 polarized cells in the S100A4-treated cultures (Figure 2D). Treatment of the cultures with the 6B12, the S100A4-neutralizing antibody, partially restored the Th1/Th2 balance in the S100A4-treated population indicating that the effect was S100A4 dependent (Figure 2E).

To confirm changes of the Th1/Th2 balance in the S100A4-treated cultures, we determined the level of IFNγ, IL4 and IL10 signature cytokines in the CM. Sandwich ELISA analyses showed a reduction of IFNγ and increase in the IL10 in the CM of S100A4-challenged T-cells (Figure 2F). The concentration of IL4 in samples was below limit necessary for detection (data not shown).

### Blocking S100A4-induced T-cell accumulation inhibits spontaneous tumor development and metastasis

The anti-S100A4 monoclonal antibody, 6B12, exhibited anti-metastatic activity by blocking T-cell infiltration in a tumor graft mouse model [24]. Injecting fully transformed cancer cells does not allow for studying the early steps of cancerogenesis. In order to develop the antibody as a potential therapeutic treatment, the assessment of its efficacy during pre-malignant steps of metastatic cancer development is especially important. For this purpose we used a PyMT-spontaneous metastatic mammary cancer mouse model [31].

**Figure 3:**
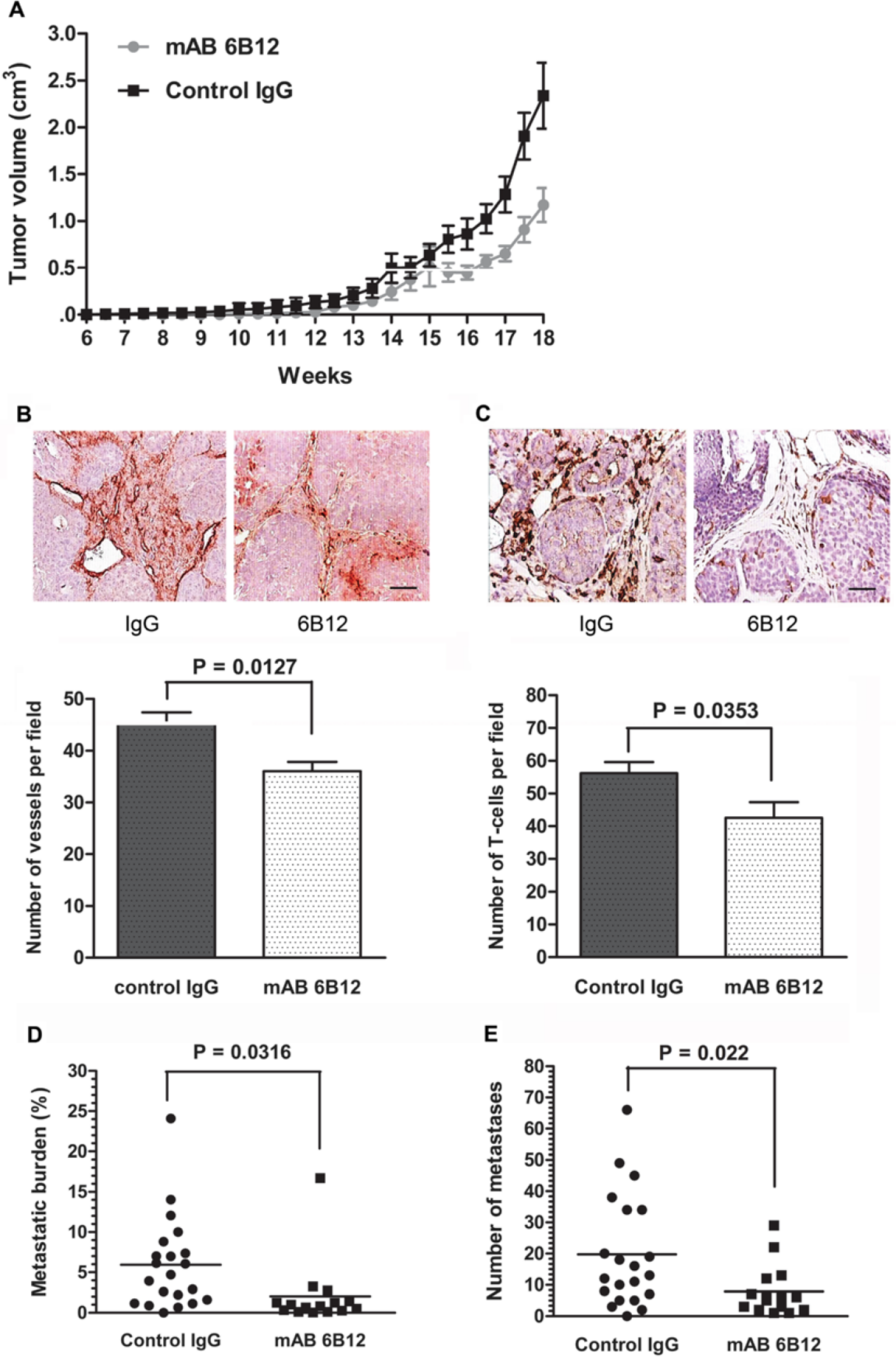
**6B12 antibody suppresses primary tumor and lung metastasis development and inhibits T-lymphocyte attraction in the PyMT model. (A)** Primary tumor growth of PyMT mice treated with 6B12 (n = 15) or IgG control (n = 21). **(B)** Upper panel: Representative images of blood vessel density in the tumor tissue sections from tumor-bearing mice treated with 6B12 and IgG (immunostaining with anti-CD31 antibody. Bar, 50 μm. Lower panel: Vessel density of primary tumors developed in PyMT mice treated with 6B12 (n = 6) or IgG control (n = 9). **(C)** Upper panel: Representative images of tumor tissue sections from 12 week-old PyMT mice treated with 6B12 antibody or with IgG control stained with anti-CD3 antibody. Bar, 50 μm. Lower panel: Quantification of CD3+ T-lymphocytes in the vicinity of primary tumor lesions from 12-week-old PyMT mice treated with 6B12 (n = 5) or IgG control (n = 8). **(D)** Metastatic burden in the lungs of PyMT mice treated with 6B12 (n = 15) or IgG control (n = 21). **(E)** Number of metastatic foci per lung per mouse in PyMT mice treated with 6B12 or IgG control.

Administration of 6B12 to PyMT tumor-bearing mice led to a delay in the development of tumors and reduction in the rate of tumor growth (Figure 3A). This delay in tumor growth could be caused by the suppression of the pro-angiogenic function of S100A4 [10,21]. We therefore determined tumor vessel density by staining tissue sections with the endothelial-specific anti-CD31 antibody. Indeed, quantifying capillaries in the tissue sections, demonstrated a substantial decrease in vessel density in tumors of 6B12-treated animals compared to the control (Figure 3B).

We next analysed whether the 6B12 antibody was able to block T-cell infiltration in pre-malignant tumors. The resulting data showed that 6B12 significantly reduced the accumulation of T-cells in primary tumors at the adenoma (MIN)/early carcinoma stage (Figure 3C).

Finally, end-point analyses of the metastatic burden as well as the overall number of metastases in the lungs of 6B12-treated mice, showed a significant reduction of both parameters when compared with the control group (Figure 3D and E). This data indicated that 6B12 was instrumental in manipulating tumor growth as well as metastasis formation when applied during early tumor development.

### The pre-metastatic niche is suppressed by the S100A4 neutralizing antibody

**Figure 4:**
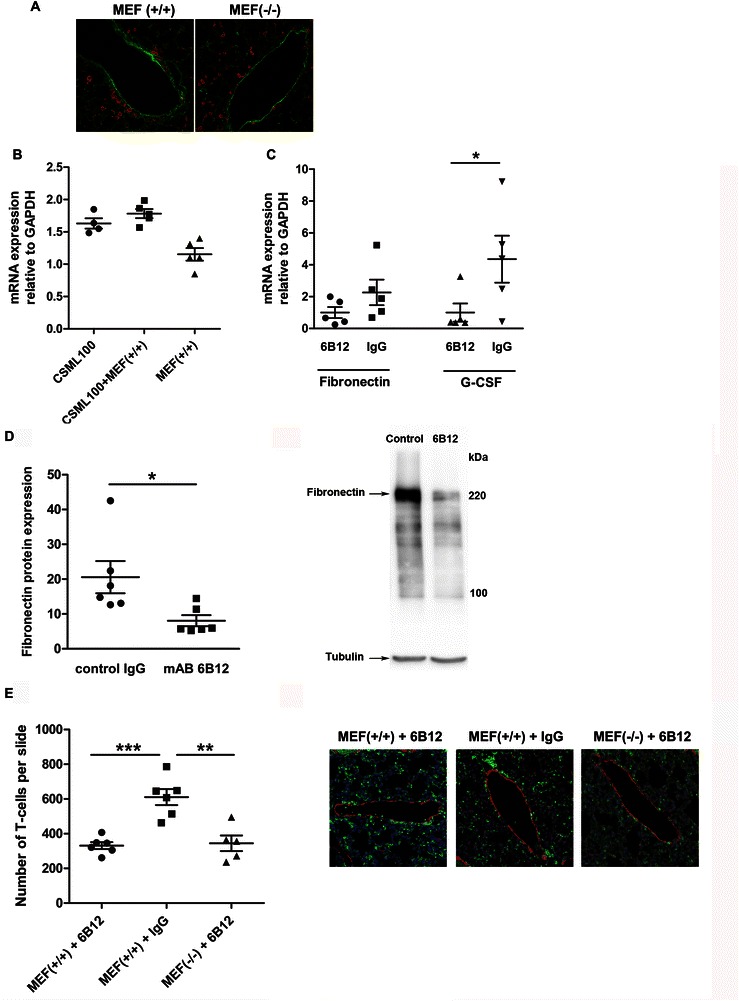
**6B12 antibody suppresses the formation of the pre-metastatic niche in the lungs.** Unless otherwise indicated, the primary tumor in mice was induced by s.c. injection of CSML100 cells followed by i.v. administration of S100A4(+/+) MEFs. **(A)** Immunohistochemical staining of pre-metastatic lung tissue sections from tumor-bearing mice boosted with S100A4(+/+) and S100A4(-/-) MEFs using antibodies against CD3 (red) and FN (green) (400x magnification). **(B)** qRT-PCR analysis of FN expression in lungs of tumor-bearing mice non-boosted or boosted with S100A4(+/+) MEFs. Mice only boosted with S100A4 (+/+) MEFs served as control. **(C)** qRT-PCR analysis of FN and G-CSF expression in the pre-metastatic lungs after treatment with the 6B12 antibodies. **(D)** Quantification of the FN protein expression level in pre-metastatic lungs of mice treated with the 6B12 antibodies (left panel). Right panel: Western-blot analysis of FN expression in pre-metastatic lungs of mice treated with 6B12 or IgG control (right panel). **(E)** Quantification of CD3+ T-cells at the site of blood vessels in pre-metastatic lungs of CSML100 tumor-bearing mice, co-injected with S100A4(+/+) or S100A4(-/-) MEFs and treated with 6B12 or IgG control (left panel). Representative images of immunofluorescence staining of pre-metastatic lungs using antibodies against CD3 (green) to visualize T-cells and smooth muscle actin (red) to visualize blood-vessels. The nucleus is stained by 4′,6-diamidino-2-phenylindole (DAPI, blue). (*, *P* < 0.05; **, *P* < 0.01; ***, *P* < 0.001, 400x magnification, right panel).

The reduced number of metastatic nodules in lungs of 6B12 treated PyMT-mice suggests that it could affect the formation of the pre-metastatic niche. We therefore analysed the efficacy of the 6B12 antibody in blocking the pre-metastatic niche by monitoring hallmarks of pre-metastatic niche formation, such as the deposition of FN, the production of G-CSF and the accumulation of T-cells [13,32,33]. Tumor-bearing mice at the pre-metastatic stage were treated with the 6B12 antibody or with the IgG control. The status of the pre-metastatic niche was analysed by immunohistochemical staining of lung tissue sections, which showed increased FN deposits as well as accumulation of T-cells around the blood vessels in pre-metastatic lungs, of mice boosted by S100A4(+/+)MEFs compared to the control S100A4(-/-)MEFs (Figure 4A). Quantitative analysis of FN transcripts in the lungs confirmed increased FN gene expression in tumor-bearing mice compared to control mice injected only with S100A4(+/+) MEFs. Inducing pre-metastatic niche formation by boosting mice with S100A4(+/+) MEFs further increased FN transcription, although the results were not statistically significant (Figure 4B). The 6B12 antibody showed a tendency to suppress FN transcription (*P* = 0.093) (Figure 4C), whereas analysis of FN protein expression showed a significant reduction of FN in the 6B12- treated group (n = 6, Figure 4D). Similar to FN, the transcription of G-CSF in pre-metastatic lungs decreased in response to 6B12 treatment (*P* = 0.033, Figure 4C). Quantifying the amount of T-cells accumulated in the vicinity of blood vessels within pre-metastatic lungs showed that the treatment with the 6B12 antibody led to a substantial reduction in T-cell numbers compared to the non-treated control (Figure 4E).

### S100A4(-/-) mice display a reduced number of T-cells in the spleen

The suppression of tumor development and metastasis in S100A4(-/-) mice was associated with a decrease in T-cell infiltration [12,13], suggesting that S100A4 could affect T-cell production in general. To explore this possibility we performed a detailed analysis of the T-cell compartment of lymphoid organs of S100A4(-/-) mice.

Analysis of thymus, spleen and lymph nodes of S100A4(+/+) and S100A4(-/-) mice (n = 12) showed that the overall cellularity of organs was similar, with a slight tendency towards less cellularity in S100A4(-/-) spleens, which was not statistically significant (Additional file 2: Table S3).

The T-cell population in the thymus of S100A4(-/-) and S100A4(+/+) mice within the double negative (DN) compartment, based on the expression of CD44 and CD25, was not affected by S100A4. Furthermore, the proportion of CD4^+^ and CD8^+^ T-cells was similar (data not shown).

**Figure 5:**
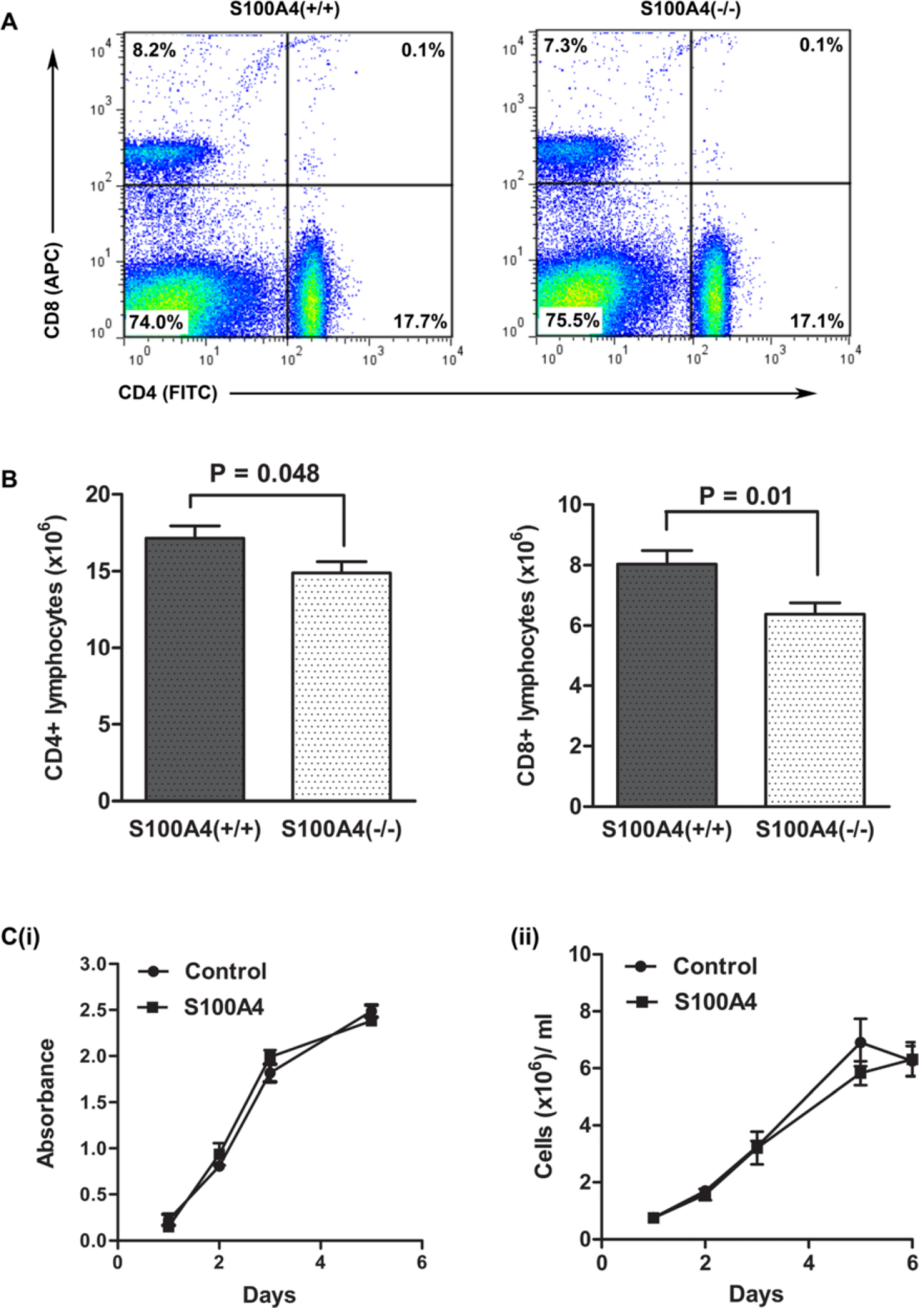
**S100A4(-/-) mice have smaller CD4**^**+**^**and CD8**^**+**^**populations in the spleen. (A)** Representative pictures of the flow-cytometry analysis of CD4^+^ and CD8^+^ T-cells from the spleen of S100A4(+/+) and S100A4(-/-) mice. Shown are the average percentages of CD4^+^ and CD8^+^ lymphocytes in the spleen (n = 12). **(B)** The absolute number of CD4^+^ and CD8^+^ lymphocytes in the spleen of S100A4(+/+) and S100A4(-/-) mice (n = 12) determined by flow-cytometry. **(C)** (i) Cell viability (LDH assay) of primary T-lymphocytes expanded with or without S100A4 protein. The LDH activity is measured at days 1, 2, 3 and 5. Proliferation rate of primary T-lymphocytes expanded with or without S100A4 protein. (ii) The number of cells is measured at days 1, 2, 3, 5 and 6.

In contrast, the absolute number of CD4^+^ and CD8^+^ T-lymphocytes in S100A4(-/-) spleens was significantly reduced compared to the control, whereas the proportion of CD8^+^ and CD4^+^ cells remained unchanged (Figure 5A,B). We did not observe any differences in the proportion of naïve (CD62L^+^CD44^-^) and memory (CD62L^-^CD44^+^) CD4^+^ T-cells, as well as naïve (CD62L^+^CD44^-^) and memory (CD62L^-^CD44^+^) CD8^+^ T-cells (data not shown).

The reduction in the absolute number of T-cells in the spleen of S100A4(-/-) mice could reflect the effect of S100A4 on the survival of T-cells. This proposition was of interest since it has been shown earlier that extracellular S100A4 stimulated survival of neurons and the viability of cardiac myocytes [18,34]. We therefore analyzed growth and survival of T-cell populations *in vitro* in the presence of S100A4 for 6 days. As shown in Figure 5C, S100A4 did not affect the viability of cells as judged by a LDH cell viability assay. The proliferation rate of T-cells in culture was also similar.

## Discussion

Metastasis is a complex process, which relies on a cross-talk between emerging cancer cells and the surrounding stroma, both at the site of the primary tumor and at the pre-metastatic niche. This suggests that targeting the microenvironment could be an efficient route in combating the metastatic disease.

Immune cells, invading the primary tumor site, are important components of the tumor stroma, indicating that inflammatory processes substantially influence tumor progression. Similar to the primary tumor, metastasis formation is also determined by the presence of a variety of inflammatory cells in the pre-metastatic organs [35].

It has been shown that, depending on the context, immune cells in the primary tumor could act as either anti- or pro-tumorigenic [36,37]. For example, in human breast cancer a CD68 ^high^/CD4 ^high^/CD8 ^low^ T-cell signature is significantly correlated with reduced patient survival [38]. Additionally, a high percentage of CD4^+^ T-cells positively correlates with tumor stage and metastasis [39]. CD4^+^ Th2-polarized T-lymphocytes stimulate pulmonary metastasis by regulating the pro-tumor properties of tumor-associated macrophages [40]. Finally, blocking macrophage recruitment in combination with chemotherapy substantially improves therapeutic outcomes in a mouse model system, proving the principle that targeting immune cells of the tumor microenvironment could be an efficient way to combat metastasis [38]. Likewise, reducing the numbers of Th2-polarized T-cells could serve as an effective method of anti-metastatic therapy, since this subpopulation of T-cells has been shown to regulate macrophage accumulation [40].

The S100A4 protein exhibits, at least in part, its pro-metastatic function as an extracellular factor produced by stroma-associated fibroblasts that attracted T-cells to the tumor site and to the pre-metastatic lungs [12,13]. Moreover, S100A4 is found to be strongly up-regulated in several different human inflammatory disorders [41-43]. Recently S100A4 has been shown to serve as a link between metastatic and pro-inflammatory pathways [25]. We therefore suggest that S100A4 in the tumor microenvironment acts as a pro-inflammatory factor that leads to modulation of T-cells and propose that neutralizing S100A4 protein activity might lead to restoration of a “normal” immune microenvironment at the site of the primary tumor and the pre-metastatic niche.

Pre-metastatic lungs, preconditioned by S100A4(+/+)MEFs, exhibited an altered cytokine repertoire that substantially overlapped with the cytokines produced by S100A4-boosted T-cells [13]. Cytokines, secreted by T-cells in response to S100A4, showed increased levels of Th2-characteristic cytokines as well as a decrease in the level of IFN-γ, suggesting relatively higher representation of Th2-polarized cells in culture. Recently, we also reported an S100A4-dependent increase of IL13 production in T-cells [41]. This indicates that S100A4 could induce alterations that lead to a shift in the Th1/Th2 polarization balance.

Moreover, S100A4 activated the Jak/Stat and MAP kinase pathways in T-cells. In fact, similar to T-cells, the S100A4 protein activated Jak3/Stat3 and MAP-kinase pathways in other cell types [17,18]. The Jak3/Stat3 and MAP-kinase pathways are known to affect T-cell differentiation [30,44-46].

Indeed, this paper has demonstrated an extracellular S100A4 induced alteration in the Th1/Th2 balance. S100A4 reduced the percentage of Th1-polarized cells leading to a shift in the Th1/Th2 ratio. The S100A4-neutralizing antibody restored it, indicating that the process was S100A4-dependent. Importantly, the S100A4 protein did not affect proliferation, survival, nor the proportion of CD4^+^ T-cells in the population, therefore suggesting that S100A4’s influence is isolated to T-cell polarization.

It has been shown that the prevalence of Th2-polarized cells in the tumor microenvironment is strongly associated with the metastatic progression of a tumor [7,40,47,48]. Based on this, we propose that S100A4-induced alterations of the Th1/Th2 polarization balance in the tumor microenvironment can therefore promote tumor progression. To reach a final conclusion on these propositions, we will need to obtain *in vivo* data that directly links S100A4 with the regulation of T-cell differentiation patterns.

The 6B12 antibody not only reduced T-cell infiltration in pre-malignant tumors and pre-metastatic lungs, but also suppressed tumor growth and vascular density, which was not observed earlier in a tumor graft model [13,24]. We suggest therefore that the pro-angiogenic activity of S100A4 unfolds at the pre-malignant stage. Recently, an anti-angiogenic effect of a different anti-S100A4 antibody was demonstrated in another model [47].

In addition, S100A4 attracted T-cells to the primary tumor and pre-metastatic lungs, suggesting that it could affect the homing of T-cells. Indeed, the number of T-cells in the spleen of S100A4-deficient mice was reduced, suggesting that it could distress homing of T-cells to secondary lymphoid organs. One could speculate that mechanisms involved S100A4-dependent attraction of T-cells to the tumor and spleen are similar.

We also suggest here that S100A4-dependent T-cell activation is essential for its pro-metastatic function. By utilizing this function S100A4 modifies the tumor microenvironment and pre-conditions secondary organs to accept tumor cells.

Current cancer treatments, including chemotherapy, have thus far had a limited effect on metastatic tumors. The data in this paper clearly suggests that blocking S100A4 activity, using the 6B12 antibody, should hamper the pro-metastatic activity of the tumor microenvironment by restoring the T-cell polarization balance. This, in combination with cytotoxic chemotherapy, could substantially increase the effectiveness of anti-cancer treatment in metastatic tumors.

## Conclusions

Treatment of metastatic disease is one of the biggest challenges in modern cancer research. Blocking activity of the pro-metastatic S100A4 protein is suggested as a promising approach in the fight against metastatic cancer. Here we show that T-cells, challenged with S100A4, shift their Th1/Th2 polarization balance towards the Th2 pro-tumorigenic phenotype. The Th1/Th2 balance is restored by 6B12, a S100A4 neutralizing antibody. Furthermore, 6B12 suppresses the attraction of T-cells to the site of the early primary tumor and pre-metastatic lungs, delays primary tumor growth and inhibits metastases. Overall our data suggests that the restoration of the T-cell polarization balance at the site of the primary tumor and the pre-metastatic niche, is a key mechanism of action of the 6B12 antibody.

## Additional files

Additional file 1:
**The ARRIVE guidelines Animal Research: Reporting In Vivo Experiments.**

Additional file 2: Table S1. List of genes up- and down-regulated in T-cells treated with S100A4. **Table S2.** List of cytokines differentially expressed in the CM from T-cells stimulated by S100A4. **Figure S1.** The relative ratio of Th1/Th2 polarized T-cells after stimulation with CD3/CD28/IL2 and S100A4 protein for 6 days. **Table S3.** The cellularity in lymphoid organs of S100A4(+/+) and S100A4(-/-) mice (n = 12).
