# Supplementary material for: S100A4-neutralizing antibody suppresses spontaneous tumor progression, pre-metastatic niche formation and alters T-cell polarization balance
Source: BMC Cancer. 2015 Feb 12;15:44. doi: 10.1186/s12885-015-1034-2 (PMC4335362; doi:10.1186/s12885-015-1034-2)
Supplement: Additional file 1: — The ARRIVE guidelines Animal Research: Reporting In Vivo Experiments. [file 12885_2015_1034_MOESM1_ESM.docx]

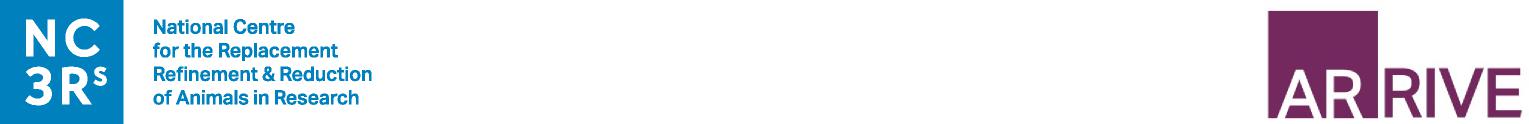


The ARRIVE guidelines Animal Research: Reporting In Vivo Experiments

Birgitte Grum-Schwensen^2^, Jörg Klingelhöfer^1^, Mette Beck^2^, Charlotte Menné Bonefeld^3^, Petra Hamerlik^2^, Per Guldberg^2^, Mariam Grigorian^1^, Eugene Lukanidin^2^, Noona Ambartsumian^1§^

*^1^Institute of Neuroscience and Pharmacology, Faculty of Health Sciences, Copenhagen University, 2200 Copenhagen, Denmark*

*^2^Danish Cancer Society Research Center, Copenhagen, 2100, Denmark*

*^3^Institute of International Health, Immunology and Microbiology, Faculty of Health Sciences, Copenhagen University, 2200 Copenhagen, Denmark*

The ARRIVE guidelines are designed to improve the reporting of animal research. This document demonstrates how the ARRIVE guidelines can be used in practice to report animal research, by providing specific examples for each point of the guidelines. The examples given are from a wide range of research using a range of animal species. Each example has been chosen for meeting the criteria of the checkpoint mentioned and does not imply the entire manuscript complies with the ARRIVE guidelines.

| ITEM RECOMMENDATION EXAMPLE |
| --- |

S100A4-neutralizing antibody suppresses spontaneous tumor progression, pre-metastatic niche formation and alters T-cell polarization balance.

Title 1 Provide as accurate and concise a description of the

content of the article as possible.

Background

Abstract 2 Provide an accurate summary of the background,

research objectives, including details of the species or strain of animal used, key methods, principal findings and conclusions of the study.

The tumor microenvironment plays a determinative role in stimulating tumor progression and metastasis. Notably, tumor-stroma signals affect the pattern of infiltrated immune cells and the profile of tumor-released cytokines. Among the known molecules that are engaged in stimulating the metastatic spread of tumor cells is the S100A4 protein. S100A4 is known as an inducer of inflammatory processes and has been shown to attract T-cells to the primary tumor and to the pre-metastatic niche. Furthermore, S100A4 affects the polarization of T-cells. The present study aims to examine the immunomodulatory role of S100A4 during tumor progression and assess the mode of action of 6B12, a S100A4 neutralizing antibody.

Methods

The therapeutic effect of the 6B12 antibody was evaluated in two different mouse models. First, in a model of spontaneous breast cancer development we assessed the dynamics of tumor growth and metastasis. For these experiments a transgenic mouse bearing Polyoma middle T oncogene under control of MMTV promoter was used. These mice develop spontaneous mammary tumors highly metastatic to the lungs [1]. 6-week-old PyMT female mice were injected with a loading dose (7.5 mg/kg in a volume of 100 μl) of 6B12, or IgG control, intra-peritoneal. Second, in a model of metastatic niche formation we determined the expression of metastatic niche markers by immunohistochemistry as well as RNA and protein expression analyses. The mouse model was described earlier in [1]. The S100A4(-/-)A/Sn mouse strain was injected s/c with highly metastatic CSML100 mammary cancer cells, followed by intravenous injection of S100A4(+/+) MEFs and injection of 6B12 antibody (7.5 mg/kg) of 6B12, or IgG control intra-peritoneally three times a week. The levels of cytokine expression were assessed using antibody as well as PCR arrays and the results confirmed by qRT-PCR and ELISA. T-cell phenotyping and *in vitro* differentiation analyses were performed by flow cytometry.

Results

We show that the S100A4 protein alters the expression of transcription factor and signal transduction pathway genes involved in the T-cell lineage differentiation. T-cells challenged with S100A4 demonstrated reduced proportion of Th1-polarized cells shifting the Th1/Th2 balance towards the Th2 pro-tumorigenic phenotype. The 6B12 antibody restored the Th1/Th2 balance. Furthermore, we provide evidence that the 6B12 antibody deploys its anti-metastatic effect at the pre-malignant stage of primary tumor development and pre-metastatic niche formation, by suppressing the attraction of T-cells to the site of primary tumor and pre-metastatic lungs. This was associated with delayed primary tumor growth, decreased vessel density and inhibition of metastases.

.

The ARRIVE guidelines. Originally published in *PLOS Biology*, June 2010^1^

|  |  | Conclusion  The S100A4 blocking antibody (6B12) reduces tumor growth and metastasis in a model of spontaneous breast cancer. The 6B12 antibody treatment inhibited T cell accumulation at the primary and pre-metastatic tumor sites. *In vitro* the 6B12 antibody blocked the S100A4 induced shift of Th1/Th2 balance towards prevalence of the tumor promoting Th2-phenotype. This suggests the antibody acts as an immunomodulatory agent and thus supports the view that the 6B12 antibody is a promising therapeutic candidate to fight cancer. |
| --- | --- | --- |
|  |  |  |
| INTRODUCTION |  | |
| Background 3 | 1. Include sufficient scientific background (including relevant references to previous work) to understand the motivation and context for the study, and explain the experimental approach and rationale. | Among the numerous molecules of the tumor microenvironment that play causal roles in metastatic spread of cancer cells is the S100A4, which belongs to the S100 family of small Ca-binding proteins. This group of proteins is characterized by both intra- and extra-cellular activity. S100A4 is expressed in many human cancers, and is correlated with poor prognosis and an elevated incidence of metastasis [2, 3]. By using transgenic and knockout mouse models stroma-cell derived S100A4 was shown to have a causal role in tumor progression [1, 4-8]. It has been suggested that it modulates the microenvironment, both at the site of the primary tumor and the pre-metastatic niche [1, 8, 9].  Tumor-associated fibroblasts are one of the sources of extracellular S100A4 in tumors [6, 8]. Pro-tumorigenic signal transduction pathways as well as the production of proteases and cytokines from various cell types are activated by S100A4 [10-13]. Furthermore, it has been shown that S100A4 acts as an angiogenic factor, as well as attracting T-cells to the site of the growing tumor and pre-metastatic lungs [1, 5, 13, 14]. Taking into account its pivotal role in metastasis, S100A4 was suggested as a potential target for a novel cancer therapy. For example, the anti-inflammatory drug sulindac, which inhibits S100A4 transcription, effectively suppressed colon cancer metastasis [15]. Recently, we have shown that the S100A4 function-blocking antibody (6B12) suppressed metastasis formation in mice grafted with metastatic mammary cancer cells. Furthermore, this study suggested that, the anti-S100A4 antibody decreased metastatic burden by blocking the attraction of T-cells [16]. *In vitro*, S100A4 was chemo-attractive for T-cells and modified the pattern of cytokines produced by these cells [1].  Based on the above, we propose that S100A4 might alter the T-cell balance in the tumor microenvironment and thereby promote cancer metastasis. We further suggest that blocking S100A4 activity can reinstate the “normal” T-cell balance and by this suppress the metastasis. |
|  |  |  |
|  | 1. Explain how and why the animal species and model being used can address the scientific objectives and, where appropriate, the study’s relevance to human biology. | The *in vivo* tumor graft and genetically modified mouse models were indispencible to study the mechanisms of metastatic spread of the tumor. For S100A4 studies both types of mouse models were used to prove its role as metastasis promoter[2, 17]. In the present study we chose two mouse models to study the mechanism of anti-metastatic activity of S100A4 function blocking antibody. |
|  |  |  |
| Objectives 4 | Clearly describe the primary and any secondary objectives of the study, or specific hypotheses being tested. | We propose that S100A4 might alter the T-cell balance in the tumor microenvironment and thereby promote cancer metastasis. We further suggest that blocking S100A4 activity can reinstate the “normal” T-cell balance and by this suppress the metastasis. |
|  |  |  |

The ARRIVE guidelines. Originally published in *PLOS Biology*, June 2010^1^

METHODS

Ethical statement 5 Indicate the nature of the ethical review permissions, relevant licences (e.g. Animal [Scientific Procedures] Act 1986), and national or institutional guidelines for the care and use of animals, that cover the research.

All mouse experiments were performed according with the charter of fundamental animal rights of the European Union (20007C364/01, Dec 7 2000). Permission to work with mouse tumor models and breeding of genetically modified animals has been granted to Noona Ambartsumian (license 2013-15-2934-00864/ACHOV).

Study design 6 For each experiment, give brief details of the study

design including:

1. The number of experimental and control groups.
2. Any steps taken to minimise the effects of subjective bias when allocating animals to treatment (e.g. randomisation procedure) and when assessing results (e.g. if done, describe who was blinded and when).
3. The experimental unit (e.g. a single animal, group or cage of animals).
4. A time-line diagram or flow chart can be useful to illustrate how complex study designs were carried out.

***PyMT mouse model***

40 virgin female PyMT mice (Polyoma-middle T spontaneous metastatic mammary cancer model) of A/Sn genetic background were used for the experiments. Breeding and genotyping was performed as described earlier [1]. 6-week-old PyMT female mice were injected either with a loading dose (7.5 mg/kg) of 6B12 (n=20), or IgG control (n=20), intra-peritoneal. Injections of antibodies were repeated three time a week. 5 animals of each experimental group were sacrificed at age of 12 weeks. For the rest animals were sacrificed and processed as described earlier when the biggest tumor reached 1 cm^3^ [1].

***Pre-metastatic assay***

(A) CSML100 metastatic mouse mammary carcinoma cells (1x10^6^) [18] were injected subcutaneously into S100A4(-/-) mice of A/Sn genetic background [5] followed by intravenous injection of 2.5x10^5^ S100A4(+/+) or S100A4(-/-) mouse embryonic fibroblasts (MEFs). Experiment comprises of 5 groups 5 mice per group. Experiment was repeated twice.

Group#1 injected CSML100 cells

Group#2 injected CSML100 + MEF(+/+)

Group#3 injected CSML100+MEF(-/-)

Group#4 injected MEF(+/+)

Group#5 injected MEF(-/-)

(B) For antibody effect analysis CSML100 cells (1x10^6^) were injected subcutaneously followed by intravenous injection of 2.5x10^5^ S100A4(+/+) or S100A4(-/-) mouse embryonic fibroblasts (MEFs) mixed with either 100 μg of 6B12, or IgG control. Then the mice were injected with a loading dose (7.5 mg/kg) of 6B12, or IgG control intra-peritoneally three times a week. Injections of MEFs mixed with antibodies were repeated three times with a one-week interval. Animals were sacrificed when the tumor reaches 5-6 mm in diameter (pre-metastatic phase) [1].

Experiment comprises of 6 groups 6 mice per group. Experiment was repeated twice.

**Group #1 –**s/c injection CSML100 cells

**Group #2 –**s/c injection CSML100 cells

**Group #3 –** s/c injection CSML100 cells + i/v injection MEF+/+/6B12

**Group #4 –** s/c injection of CSML100 cells + i/v injection of MEF+/+/IgG

**Group #5** s/c injection of CSML100 cells + i/v injection MEF-/-/6B12

**Group #6** s/c injection of CSML100 cells + i/v injection of MEF-/-/IgG

Timeline:

| **Day1** | **Day7** | **Day14** | **Day app 30** |  |  |
| --- | --- | --- | --- | --- | --- |
| s/c CSML100 cells |  |  | Timor size 5-6 mm | Termination of the experiment |  |
| i/v  MEF | i/v  MEF | i/v  MEF |  |  |  |

S100A4(-/-) A/Sn mice

| Day | 1 | 1 | 1. | 3. | 6. | 6. | 8. | 11. | 13. | 13 | 15. | 18. | 20 |
| --- | --- | --- | --- | --- | --- | --- | --- | --- | --- | --- | --- | --- | --- |
|  | Sc injec | Iv inject | Ip | Ip | Ip | Iv inject | Ip | Ip | Ip | Iv inject | Ip | Ip | sacrifice |
|  | CSML 100 | MEF/6B12 | 6B12 | 6B12 | 6B12 | MEF | 6B12 | 6B12 | 6B12 | MEF | 6B12 | 6B12 | * |
|  | CSML 100 | MEF/IgG | IgG | IgG | IgG | MEF | IgG | IgG | IgG | MEF | IgG | IgG | * |

The ARRIVE guidelines. Originally published in *PLOS Biology*, June 2010^1^

| Experimental procedures | 7 For each experiment and each experimental group,  including controls, provide precise details of all  procedures carried out. For example: |
| --- | --- |

1. How (e.g. drug formulation and dose, site and route of administration, anaesthesia and analgesia used [including monitoring], surgical procedure, method of euthanasia). Provide details of any specialist equipment used, including supplier(s).
2. When (e.g. time of day).
3. Where (e.g. home cage, laboratory, water maze
4. Why /e.g rationale for choice of specific anaesthetic, route of administration, drug dose used).

PyMT mouse model: 6-week-old PyMT female mice were injected either with a loading dose (7.5 mg/kg) of 6B12, or IgG control, intra-peritoneal.

Injections of antibodies were repeated three times a week. Pre-metastatic niche model: CSML100 metastatic mouse mammary carcinoma cells (1x10^6^) were injected subcutaneously with 27G needles, into S100A4(-/-) mice of A/Sn genetic background ,followed by intravenous injection with 27G needles of 2.5x10^5^ S100A4(+/+) or S100A4(-/-) mouse embryonic fibroblasts (MEFs).

For antibody effect analysis CSML100 cells (1x10^6^) were injected subcutaneously followed by intravenous injection of 2.5x10^5^ S100A4(+/+) or S100A4(-/-) mouse embryonic fibroblasts (MEFs) mixed with either 100 μg of 6B12, or IgG control. Then the mice were injected with a loading dose (7.5 mg/kg in 100µl ) of 6B12, or IgG control intra-peritoneally with 26G needles three times a week. Injections of MEFs mixed with antibodies were repeated three times with a one-week interval. Animals were sacrificed when the tumor reaches 5-6 mm in diameter (pre-metastatic phase) [1].

:

Experimental animals

8 a. Provide details of the animals used, including species, strain, sex, developmental stage (e.g. mean or median age plus age range) and weight (e.g. mean or median weight plus weight range).

b. Provide further relevant information such as the source of animals, international strain nomenclature, genetic modification status (e.g. knock-out or transgenic), genotype, health/immune status, drug or test naïve, previous procedures, etc.

Male and female S100A4(-/-) A/Sn mice aged 8-12 weeks with average weight 26g were used in the experiments (n=122) [5].

PyMT transgenic mice of the A/Sn genetic background (PyMT+/-) were used for the experiments [1]. Animals were maintained and bred for at least 15 generations in the Danish Cancer Society Research Center Animal Facility. Mice were free of known viral, bacterial and parasitic pathogens (health reports held in the DCSRC animal facility).

The ARRIVE guidelines. Originally published in *PLOS Biology*, June 2010^1^

| Housing and husbandry | 9 Provide details of:   1. Housing (type of facility e.g. specific pathogen free [SPF]; type of cage or housing; bedding material; number of cage companions; tank shape and material etc. for fish). 2. Husbandry conditions (e.g. breeding programme, light/dark cycle, temperature, quality of water etc for fish, type of food, access to food and water, environmental enrichment). 3. Welfare-related assessments and interventions that were carried out prior to, during, or after the experiment. |
| --- | --- |

All mice at DCSRC are kept under the same standard conditions:

- Housing in Scantainers
- Feed (Altromin pellets, Altromin GmbH, Germany) ad libitum
- Water (tap water with an addition of citric acid until pH 4) ad libitum*
- Light/darkness, 12 hours/12 hours (lights on 6 am - 6 pm)
- Temperature 21-24 °C
- Humidity 55 +/- 10%
- Type IIL (530 cm^2^)  or type III (825 cm^2^) polysulfon (clear) cages with wire lid
- Bedding (Tapvei wooden chips)
- Nest material (Sizzle Nest/Nestlets and/or Coccoons)
- Hiding (PVC tubes/PVC houses)
- Gnawing sticks (Tapvei)
- Oat (common fodder oat, radiated) in the bedding at changing

Cages are changed twice a week. Water is changed once a week.

All materiel is washed in the cage washer and autoclaved. It is also subjected to UV radiation at least 12 hours before use.

All experimental mice are group housed, maximum 5 animals per cage.

The facility is doing health monitoring according to the FELASA guideline and are free from these pathogens.

All animals are health checked daily including weekends and public holidays. If mice has tumors they are measured at least twice a week and mice are sacrificed when tumors reach 12 mm or if they get wounds on the tumor or have any clinical signs of illness or distress due to the tumor burden.

Sample size 10 a. Specify the total number of animals used in each

experiment, and the number of animals in each experimental group.

1. Explain how the number of animals was arrived at. Provide details of any sample size calculation used.
2. Indicate the number of independent replications of each experiment, if relevant

122 S100A4(-/-) male and female mice were used for the pre-metastatic niche experimental model. Animals were divided into 5 or 6 groups (described in details above). The group size was 5 or 6 animals per group.

40 healthy PyMT female animals were divided into 2 groups. The group size was calculated using G power software [19]

The pre-metastatic niche model experiments were repeated twice.

The pre-metastatic niche experiments to test blocking of the pre-metastatic niche with the 6B12 antibody were repeated twice.

11 a. Give full details of how animals were allocated to

experimental groups, including randomisation or matching if done.

Allocating animals to experimental groups

Animals were allocated into groups according to their bodyweight to avoid size differences between the groups

The ARRIVE guidelines. Originally published in *PLOS Biology*, June 2010^1^

|  |  | b. Describe the order in which the animals in the different experimental groups were treated and assessed. | The groups were treated in order (group 1 first, then group 2…..) |
| --- | --- | --- | --- |
|  |  |  |  |
| Experimental outcomes | 12 | Clearly define the primary and secondary experimental outcomes assessed (e.g. cell death, molecular markers, behavioural changes). | For the PyMT transgenic mouse model the primary outcome was the size of the primary tumor. Animals were sacrificed when the primary tumor reached the size of 1 cm^3^.  For the pre-metastatic niche model animals were sacrificed when the grafted tumor reached the size of 0,5-0,6 cm^3^ (pre-metastatic phase). |
|  |  |  |  |
| Statistical methods | 13 | 1. Provide details of the statistical methods used for each analysis. 2. Specify the unit of analysis for each dataset (e.g. single animal, group of animals, single neuron). 3. Describe any methods used to assess whether the data met the assumptions of the statistical approach. | The confidence level was calculated using paired or unpaired Student’s *t* test, depending on the content of the experiment, using GraphPad Prism software. Data is shown as mean ± SEM. |
|  |  |  | For each test, the experimental unit was an individual animal. |
|  |  |  |  |
|  |  |  |  |
| RESULTS |  |  | |
| Baseline data | 14 | For each experimental group, report relevant characteristics and health status of animals (e.g. weight, microbiological status, and drug or test naïve) prior to treatment or testing. (This information can often be tabulated). | The animals’ health status was monitored throughout the experiments by a health surveillance program according to Federation of European Laboratory Animal Science Associations (FELASA) guidelines. The mice were free of all viral, bacterial, and parasitic pathogens listed in the FELASA recommendations. |
|  |  |  |  |
| Numbers analysed | 15 | a. Report the number of animals in each group included in each analysis. Report absolute numbers (e.g. 10/20, not 50%^2^). | In the PyMT mouse model all the animals developed tumors (40/40).  In the pre-metastatic niche model all the animals with injected CSML100 cells developed tumors (5-6 mm).  Mice injected with MEF cells only did not develop tumors and were healthi at the time of termination of the experiment (102/122). |
|  |  |  |  |

The ARRIVE guidelines. Originally published in *PLOS Biology*, June 2010^1^

All the animals used in experiments were included into analysis.

b. If any animals or data were not included in the analysis, explain why.

.6B12 antibody suppresses primary tumor and lung metastasis development and inhibits T-lymphocyte attraction in the PyMT model. (A) Primary tumor growth of PyMT mice treated with 6B12 (n = 15) or IgG control (n = 15).

Outcomes and 16 Report the results for each analysis carried out,

estimation with a measure of precision (e.g. standard error or confidence interval).

.


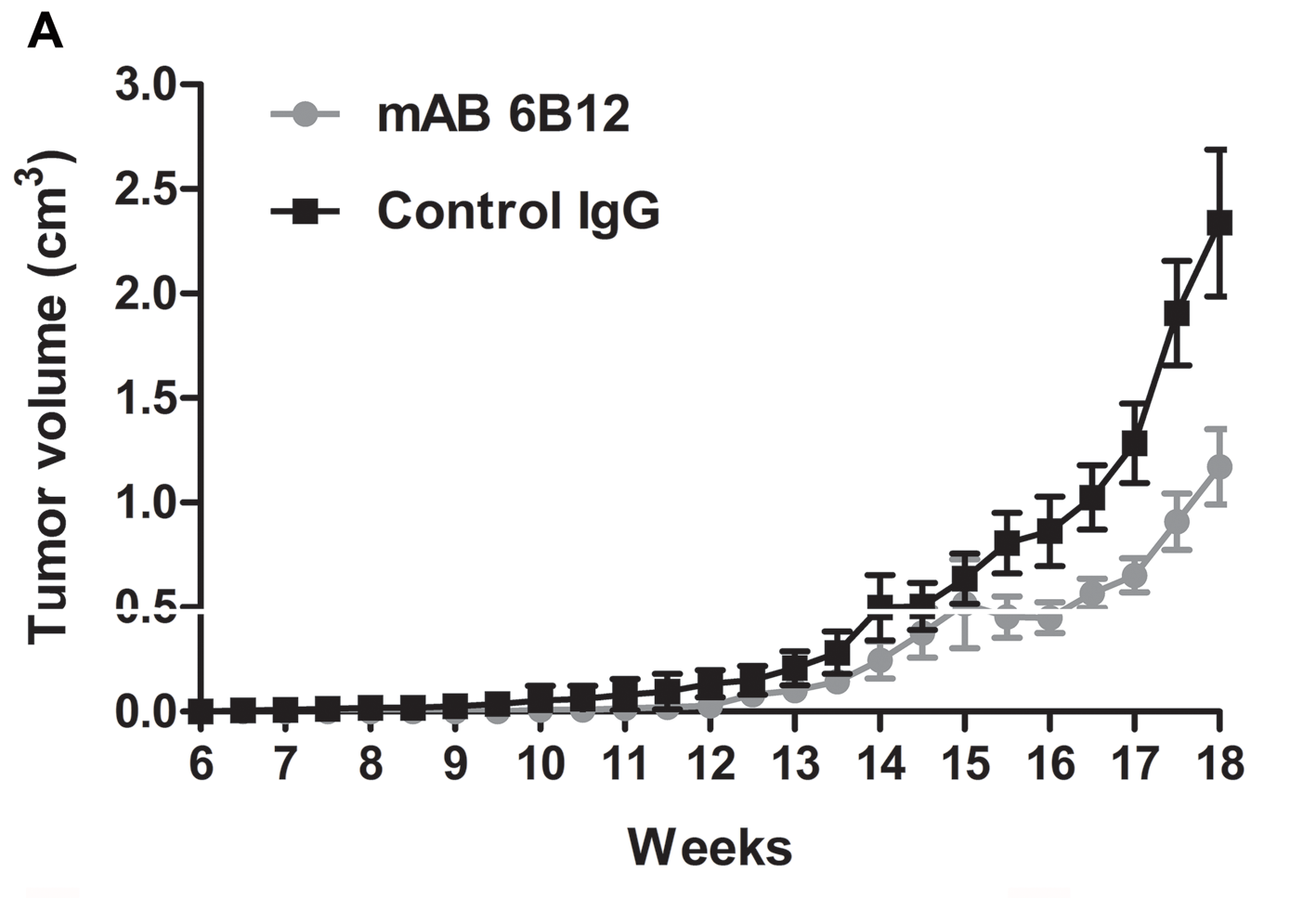


We did not observe any important adverse events in out experiments

Adverse events 17 a. Give details of all important adverse events in

each experimental group.

b. Describe any modifications to the experimental protocols made to reduce adverse events.

The ARRIVE guidelines. Originally published in *PLOS Biology*, June 2010^1^

DISCUSSION

18 a. Interpret the results, taking into account the study objectives and hypotheses, current theory

Interpretation/ scientific implications

and other relevant studies in the literature.

1. Comment on the study limitations including any potential sources of bias, any limitations of the animal model, and the imprecision associated with the results^2^.
2. Describe any implications of your experimental methods or findings for the replacement, refinement or reduction (the 3Rs) of the use of animals in research.

We suggest in this work that S100A4 in the tumor microenvironment acts as a pro-inflammatory factor that leads to modulation of T-cells and propose that neutralizing S100A4 protein activity might lead to restoration of a “normal” immune microenvironment at the site of the primary tumor and the pre-metastatic niche.

Pre-metastatic lungs, preconditioned by S100A4(+/+)MEFs, exhibited an altered cytokine repertoire that substantially overlapped with the cytokines produced by S100A4-boosted T-cells [1]. Cytokines, secreted by T-cells in response to S100A4, showed increased levels of Th2-characteristic cytokines as well as a decrease in the level of IFN-γ, suggesting relatively higher representation of Th2-polarized cells in culture. Recently, we also reported an S100A4-dependent increase of IL13 production in T-cells [20]. This indicates that S100A4 could induce alterations that lead to a shift in the Th1/Th2 polarization balance.

Indeed, this paper has demonstrated an extracellular S100A4 induced alteration in the Th1/Th2 balance. S100A4 reduced the percentage of Th1-polarized cells leading to a shift in the Th1/Th2 ratio. The S100A4-neutralizing antibody restored it, indicating that the process was S100A4-dependent. It has been shown that the prevalence of Th2-polarized cells in the tumor microenvironment is strongly associated with the metastatic progression of a tumor [21-24]. Based on this, we propose that S100A4-induced alterations of the Th1/Th2 polarization balance in the tumor microenvironment can therefore promote tumor progression. The 6B12 antibody not only reduced T-cell infiltration in pre-malignant tumors and pre-metastatic lungs, but also suppressed tumor growth and vascular density, which was not observed earlier in a tumor graft model [1, 16].

In addition, S100A4 attracted T-cells to the primary tumor and pre-metastatic lungs, suggesting that it could affect the homing of T-cells.

Current cancer treatments, including chemotherapy, have thus far had a limited effect on metastatic tumors. The data in this paper clearly suggests that blocking S100A4 activity, using the 6B12 antibody, should hamper the pro-metastatic activity of the tumor microenvironment by restoring the T-cell polarization balance.

To reach a final conclusion on these propositions, we will need to obtain *in vivo* data that directly links S100A4 with the regulation of T-cell differentiation patterns.

Generalisability/ translation

19 Comment on whether, and how, the findings of this

study are likely to translate to other species or systems, including any relevance to human biology.

Current cancer treatments, including chemotherapy, have thus far had a limited effect on metastatic tumors. The data in this paper clearly suggests that blocking S100A4 activity, using the 6B12 antibody, should hamper the pro-metastatic activity of the tumor microenvironment by restoring the T-cell polarization balance. This, in combination with cytotoxic chemotherapy, could substantially increase the effectiveness of anti-cancer treatment in metastatic tumors.

)

We acknowledge funding provided by the European Union (TuMIC, Health-F2-2008-201662), INARMERA (FP7-INCO-2010-6) and the Danish Cancer Society. The 6B12 antibody applications are covered by patent No: WO/2014/068300.

Funding 20 List all funding sources (including grant number)

and the role of the funder(s) in the study.

13)


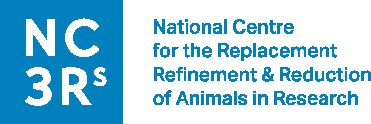


Reference List

1. Grum-Schwensen B, Klingelhofer J, Grigorian M, Almholt K, Nielsen BS, Lukanidin E, Ambartsumian N: **Lung metastasis fails in MMTV-PyMT oncomice lacking S100A4 due to a T-cell deficiency in primary tumors.** *Cancer Res* 2010, **70:**936-947.

2. Boye K, Maelandsmo GM: **S100A4 and metastasis: a small actor playing many roles.** *The American journal of pathology* 2010, **176:**528-535.

3. Mishra SK, Siddique HR, Saleem M: **S100A4 calcium-binding protein is key player in tumor progression and metastasis: preclinical and clinical evidence.** *Cancer metastasis reviews* 2012, **31:**163-172.

4. Ambartsumian N, Klingelhofer J, Grigorian M, Christensen C, Kriajevska M, Tulchinsky E, Georgiev G, Berezin V, Bock E, Rygaard J, et al: **The metastasis-associated Mts1(S100A4) protein could act as an angiogenic factor.** *Oncogene* 2001, **20:**4685-4695.

5. El-Naaman C, Grum-Schwensen B, Mansouri A, Grigorian M, Santoni-Rugiu E, Hansen T, Kriajevska M, Schafer BW, Heizmann CW, Lukanidin E, Ambartsumian N: **Cancer predisposition in mice deficient for the metastasis-associated Mts1(S100A4) gene.** *Oncogene* 2004, **23:**3670-3680.

6. Grum-Schwensen B, Klingelhofer J, Berg CH, El-Naaman C, Grigorian M, Lukanidin E, Ambartsumian N: **Suppression of tumor development and metastasis formation in mice lacking the S100A4(mts1) gene.** *Cancer Res* 2005, **65:**3772-3780.

7. Xue C, Plieth D, Venkov C, Xu C, Neilson EG: **The gatekeeper effect of epithelial-mesenchymal transition regulates the frequency of breast cancer metastasis.** *Cancer Res* 2003, **63:**3386-3394.

8. O'Connell JT, Sugimoto H, Cooke VG, MacDonald BA, Mehta AI, LeBleu VS, Dewar R, Rocha RM, Brentani RR, Resnick MB, et al: **VEGF-A and Tenascin-C produced by S100A4+ stromal cells are important for metastatic colonization.** *ProcNatlAcadSciUSA* 2011, **108:**16002-16007.

9. Forst B, Hansen MT, Klingelhofer J, Moller HD, Nielsen GH, Grum-Schwensen B, Ambartsumian N, Lukanidin E, Grigorian M: **Metastasis-inducing S100A4 and RANTES cooperate in promoting tumor progression in mice.** *PLoSOne* 2010, **5:**e10374.

10. Boye K, Grotterod I, Aasheim HC, Hovig E, Maelandsmo GM: **Activation of NF-kappaB by extracellular S100A4: analysis of signal transduction mechanisms and identification of target genes.** *IntJCancer* 2008, **123:**1301-1310.

11. Dmytriyeva O, Pankratova S, Owczarek S, Sonn K, Soroka V, Ridley CM, Marsolais A, Lopez-Hoyos M, Ambartsumian N, Lukanidin E, et al: **The metastasis-promoting S100A4 protein confers neuroprotection in brain injury.** *NatCommun* 2012, **3:**1197.

12. Grotterod I, Maelandsmo GM, Boye K: **Signal transduction mechanisms involved in S100A4-induced activation of the transcription factor NF-kappaB.** *BMCCancer* 2010, **10:**241.

13. Schmidt-Hansen B, Klingelhofer J, Grum-Schwensen B, Christensen A, Andresen S, Kruse C, Hansen T, Ambartsumian N, Lukanidin E, Grigorian M: **Functional significance of metastasis-inducing S100A4(Mts1) in tumor-stroma interplay.** *JBiolChem* 2004, **279:**24498-24504.

14. Schmidt-Hansen B, Ornas D, Grigorian M, Klingelhofer J, Tulchinsky E, Lukanidin E, Ambartsumian N: **Extracellular S100A4(mts1) stimulates invasive growth of mouse endothelial cells and modulates MMP-13 matrix metalloproteinase activity.** *Oncogene* 2004, **23:**5487-5495.

15. Stein U, Arlt F, Smith J, Sack U, Herrmann P, Walther W, Lemm M, Fichtner I, Shoemaker RH, Schlag PM: **Intervening in beta-catenin signaling by sulindac inhibits S100A4-dependent colon cancer metastasis.** *Neoplasia* 2011, **13:**131-144.

16. Klingelhofer J, Grum-Schwensen B, Beck MK, Knudsen RS, Grigorian M, Lukanidin E, Ambartsumian N: **Anti-S100A4 antibody suppresses metastasis formation by blocking stroma cell invasion.** *Neoplasia* 2012, **14:**1260-1268.

17. Ambartsumian N, Grigorian M, Lukanidin E: **Genetically modified mouse models to study the role of metastasis-promoting S100A4(mts1) protein in metastatic mammary cancer.** *JDairy Res* 2005, **72 Spec No:**27-33.

18. Ebralidze A, Tulchinsky E, Grigorian M, Afanasyeva A, Senin V, Revazova E, Lukanidin E: **Isolation and characterization of a gene specifically expressed in different metastatic cells and whose deduced gene product has a high degree of homology to a Ca2+-binding protein family.** *Genes Dev* 1989, **3:**1086-1093.

19. Faul F, Erdfelder E, Lang AG, Buchner A: **G*Power 3: a flexible statistical power analysis program for the social, behavioral, and biomedical sciences.** *Behavior research methods* 2007, **39:**175-191.

20. Bruhn S, Fang Y, Barrenas F, Gustafsson M, Zhang H, Konstantinell A, Kronke A, Sonnichsen B, Bresnick A, Dulyaninova N, et al: **A Generally Applicable Translational Strategy Identifies S100A4 as a Candidate Gene in Allergy.** *SciTranslMed* 2014, **6:**218ra214.

21. Ruffell B, Denardo DG, Affara NI, Coussens LM: **Lymphocytes in cancer development: polarization towards pro-tumor immunity.** *Cytokine Growth Factor Rev* 2010, **21:**3-10.

22. Denardo DG, Barreto JB, Andreu P, Vasquez L, Tawfik D, Kolhatkar N, Coussens LM: **CD4(+) T cells regulate pulmonary metastasis of mammary carcinomas by enhancing protumor properties of macrophages.** *Cancer Cell* 2009, **16:**91-102.

23. Hernandez JL, Padilla L, Dakhel S, Coll T, Hervas R, Adan J, Masa M, Mitjans F, Martinez JM, Coma S, et al: **Therapeutic targeting of tumor growth and angiogenesis with a novel anti-S100A4 monoclonal antibody.** *PLoSOne* 2013, **8:**e72480.

24. Liao D, Luo Y, Markowitz D, Xiang R, Reisfeld RA: **Cancer associated fibroblasts promote tumor growth and metastasis by modulating the tumor immune microenvironment in a 4T1 murine breast cancer model.** *PLoSOne* 2009, **4:**e7965.
