# Supplementary material for: S100A4-neutralizing antibody suppresses spontaneous tumor progression, pre-metastatic niche formation and alters T-cell polarization balance
Source: BMC Cancer. 2015 Feb 12;15:44. doi: 10.1186/s12885-015-1034-2 (PMC4335362; doi:10.1186/s12885-015-1034-2)
Supplement: Additional file 2: Table S1. — List of genes up- and down-regulated in T-cells treated with S100A4. Table S2. List of cytokines differentially expressed in the CM from T-cells stimulated by S100A4. Figure S1. The relative ratio of Th1/Th2 polarized T-cells after stimulation with CD3/CD28/IL2 and S100A4 protein for 6 days. Table S3. The cellularity in lymphoid organs of S100A4(+/+) and S100A4(-/-) mice (n = 12). [file 12885_2015_1034_MOESM2_ESM.docx]

Additional file**:**

1. Profiler PCR microarray analysis of T cells activated by S100A4 treatment in culture. **Table S1. List of genes up- and down-regulated in T-cells treated with S100A4.**

| **Up-regulated genes** | | **Down-regulated genes** | |
| --- | --- | --- | --- |
| **Gene** | **Fold** | **Gene** | **Fold** |
| Il10 | 1,24 | Il13 | 0,52 |
| Jak1 | 1,27 | Ccr2 | 0,58 |
| Tnf | 1,27 | Nfatc2ip | 0,66 |
| Il2ra | 1,3 | Socs5 | 0,66 |
| Inha | 1,34 | Socs1 | 0,71 |
| Gfi1 | 1,35 | Nfatc1 | 0,72 |
| Cd80 | 1,39 | Crebbp | 0,74 |
| Irf1 | 1,39 | Gata3 | 0,75 |
| Ctla4 | 1,44 | Ccr10 | 0,75 |
| Stat1 | 1,48 | Il1r1 | 0,75 |
| Il5 | 1,6 | Nfatc2 | 0,75 |
| Socs3 | 1,6 | Irf4 | 0,76 |
| Cd40 | 1,62 | Tlr4 | 0,78 |
| Tyk2 | 1,64 | Il15 | 0,8 |
| Jak3 | 1,65 | Tbx21 | 0,81 |
| Il23a | 1,98 | Ccr3 | 0,82 |
| Il17a | 2,2 | Ccr4 | 0,82 |
| Il2 | 2,21 | Tgfb3 | 0,84 |
| Il6 | 2,4 |  |  |
| Ccl7 | 2,47 |  |  |
| Csf2 | 2,51 |  |  |
| Spp1 | 3,8 |  |  |

1. Mouse cytokine antibody array analysis detecting differential expression of 22 cytokines in the conditioned media from T-cells stimulated by S100A4.

**Table S2. List of cytokines differentially expressed in the CM from T-cells stimulated by S100A4.**

| Cytokine | fold up/down-regulation |
| --- | --- |
| GCSF | 28,7 |
| GMCSF | 4,4 |
| IL2 | 2,8 |
| IL3 | NM^*^ |
| IL4 | 0,7 |
| Il5 | NM^*^ |
| IL6 | 10,6 |
| IL9 | 1,6 |
| IL10 | 1,9 |
| IL12p40p70 | 2,13 |
| IL12p70 | 1,7 |
| IL13 | 1,7 |
| IL17 | 1 |
| IFNγ | 0,6 |
| MCP-1 | 1 |
| MCP-5 | 1,7 |
| RANTES | 3,9 |
| SCF | NM^*^ |
| sTNFR1 | 2 |
| TNF-α | NM^*^ |
| Thrombopoietin | 1,4 |
| VEGF | NM^*^ |
|  |  |
| *not measurable |  |
|  |  |

1. **Figure S1. The relative ratio of Th1/Th2 polarized T-cells after stimulation with CD3/CD28/IL2 and S100A4 protein for 6 days.**

1. **Table S3.**

**The cellularity in lymphoid organs of S100A4(+/+) and S100A4(-/-) mice (n = 12).**

Total number of cells (x10^6^) in:

| Genotype | thymus | spleen | lymph nodes |
| --- | --- | --- | --- |
| S100A4(+/+) | 62.0 ± 30.4 | 98.5 ± 18.9 | 5.7 ± 1.9 |
| S100A4(-/-) | 77.1 ± 50.3 | 88.3 ± 12.9 | 6.3 ± 1.7 |
